# Supplementary figures and images for: Gene regulatory network inference in soybean upon infection by Phytophthora sojae
Source: PLoS One. 2023 Jul 7;18(7):e0287590. doi: 10.1371/journal.pone.0287590 (PMC10328377; doi:10.1371/journal.pone.0287590)

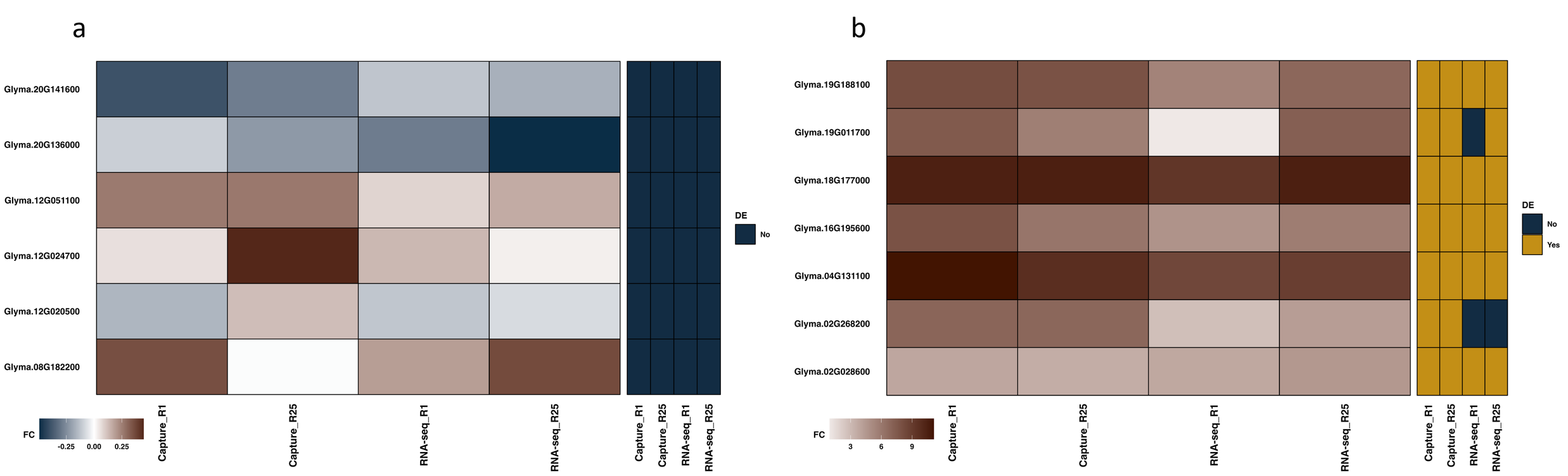

Supplement: S1 Fig — (a) Heatmap of reference gene expression in RNA- and Capture-seq. (b) Heatmap of pathogen-induced gene expression. (TIF) [file pone.0287590.s001.tif]

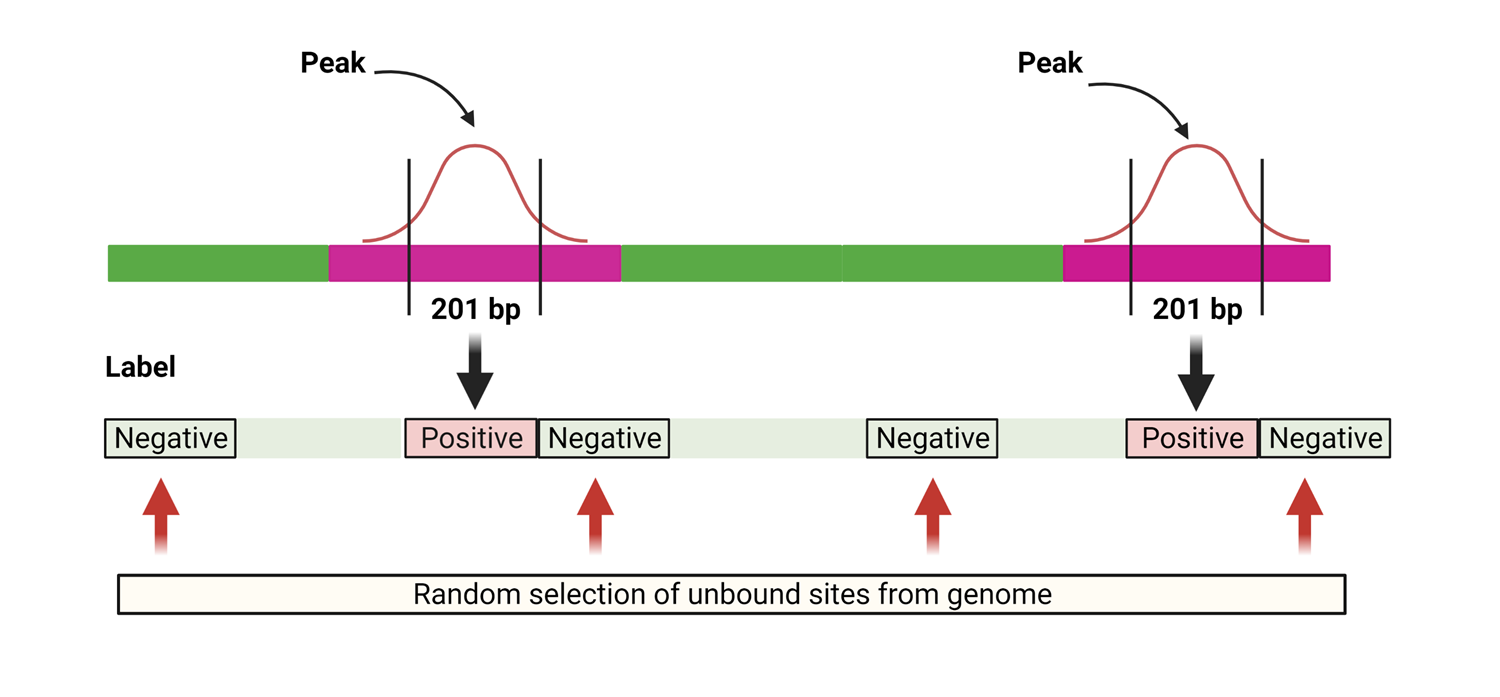

Supplement: S2 Fig — (TIF) [file pone.0287590.s002.tif]

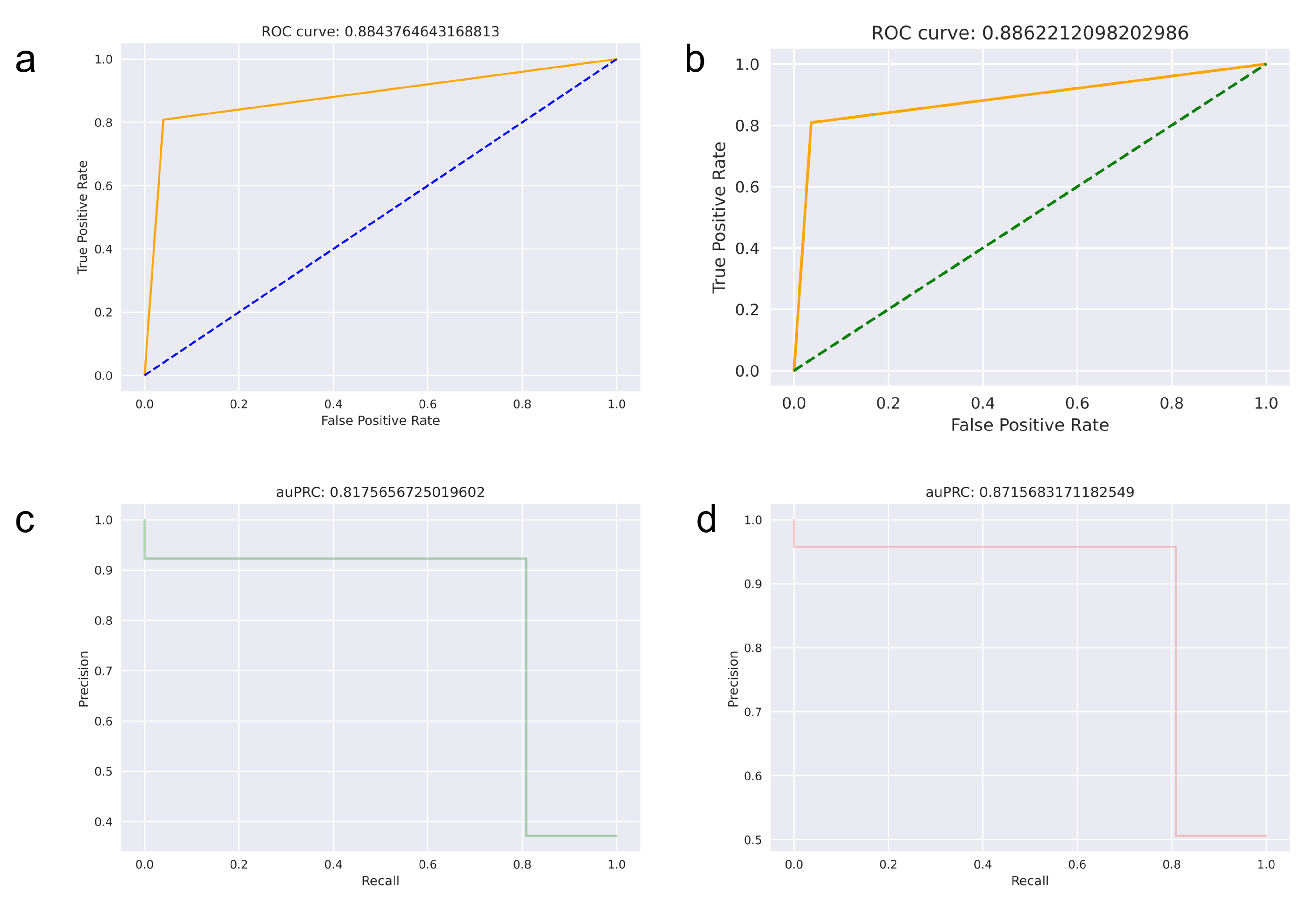

Supplement: S3 Fig — (a) auROC curve for GmWRKY30 CRNN. (b) auROC curve for GmRAV CRNN. (c) auPRC curve for GmWRKY30 CRNN. (d) auPRC curve for GmRAV CRNN. (TIF) [file pone.0287590.s003.tif]

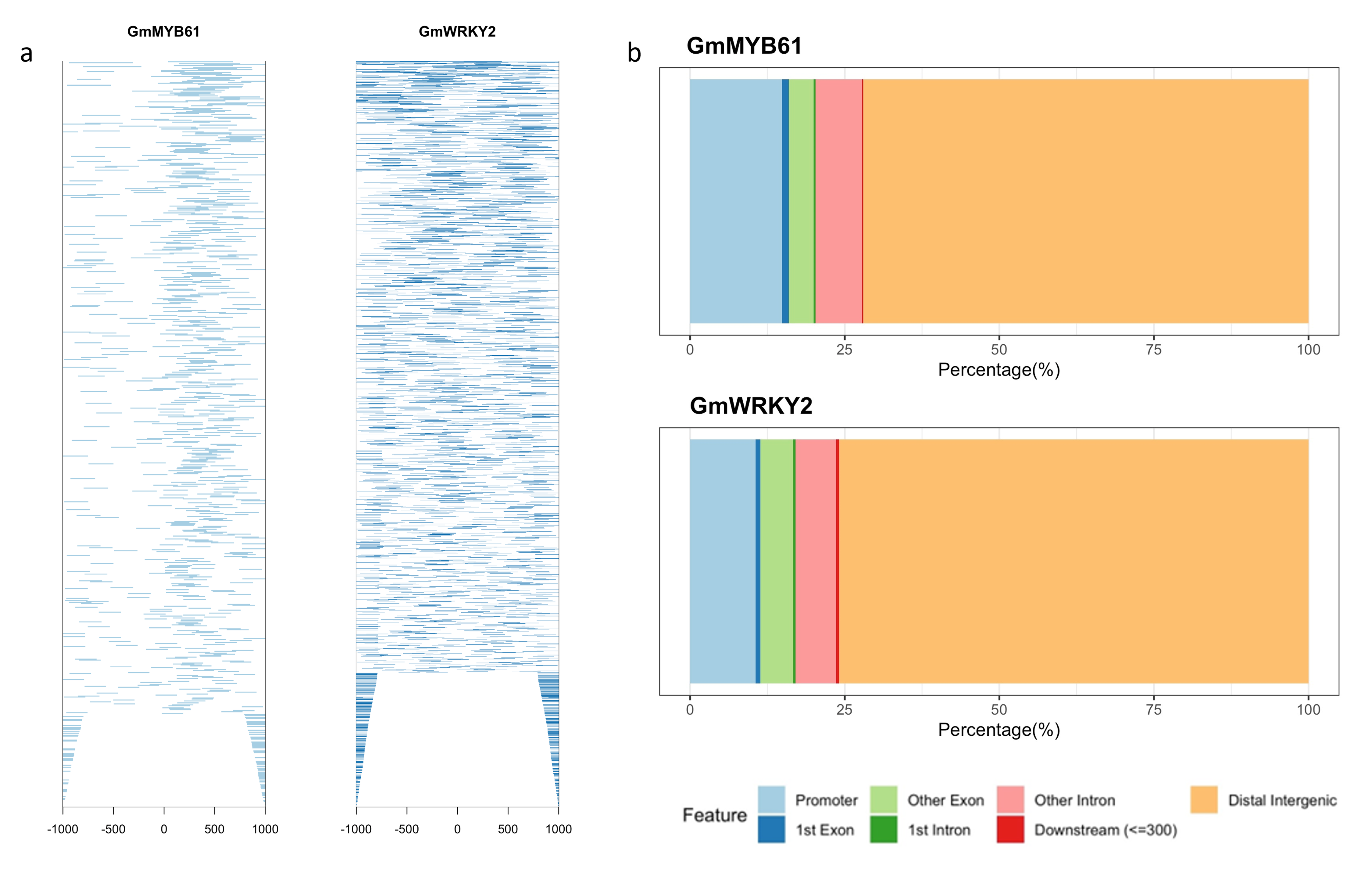

Supplement: S4 Fig — (a) Heatmap of DAP peak binding within 1,000 bp of the TSS region. (b) Distribution of DAP peaks across genomic features. (TIF) [file pone.0287590.s004.tif]

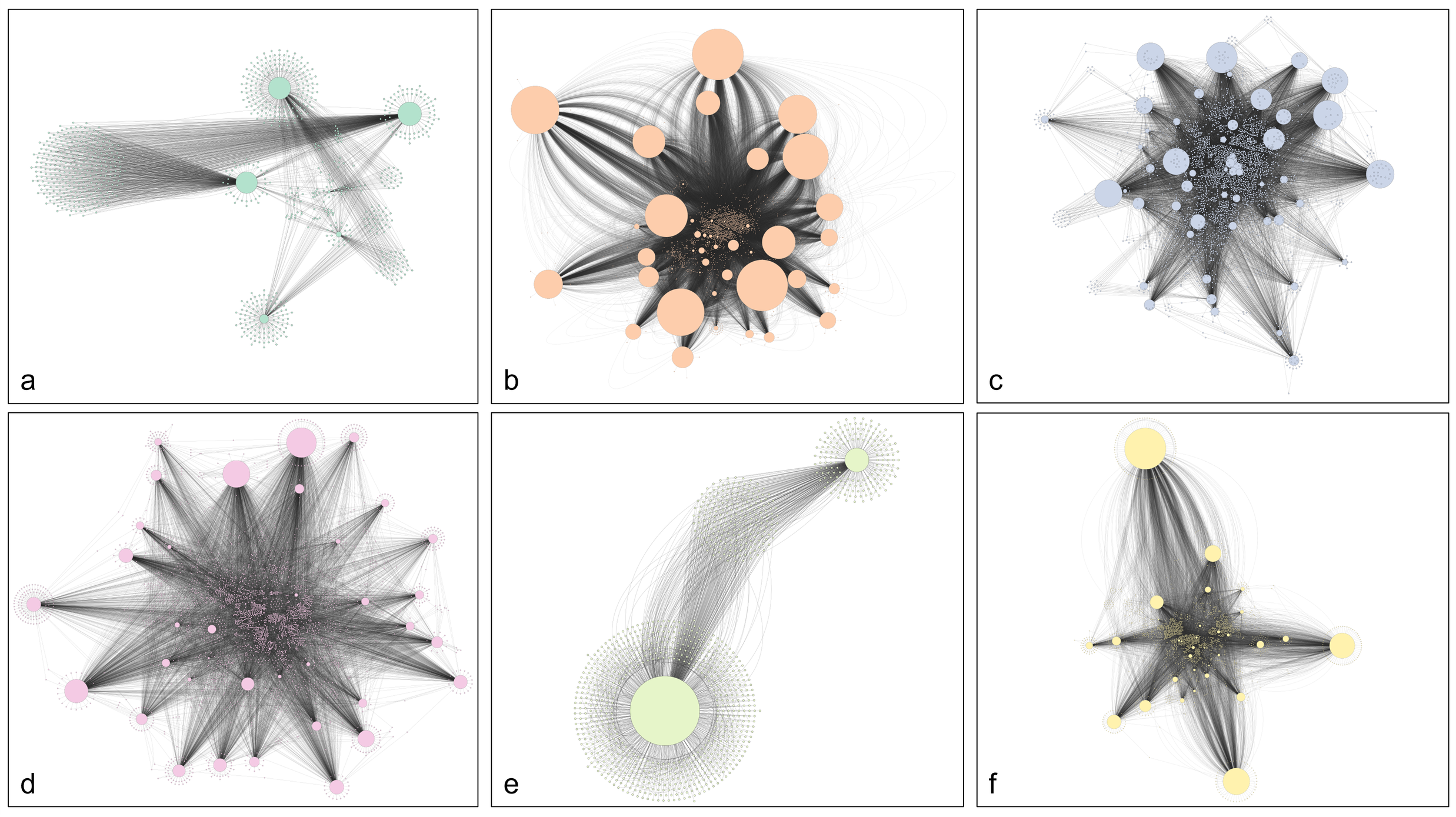

Supplement: S5 Fig — (a) bHLH GRN. (b) ERF GRN. (c) MYB GRN. (d) NAC GRN. (e) RAV GRN. (f) WRKY GRN. (TIF) [file pone.0287590.s005.tif]

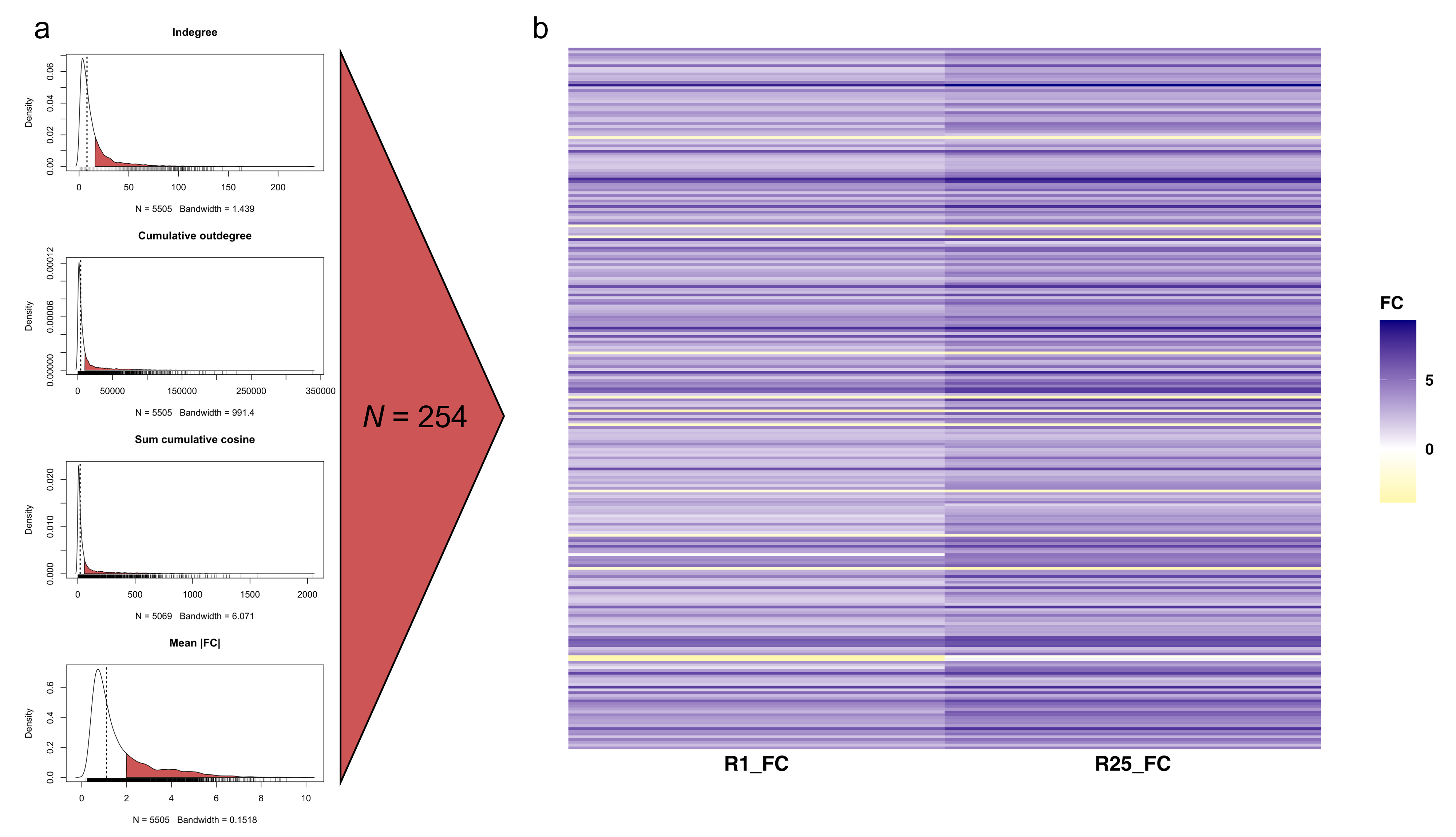

Supplement: S6 Fig — (a) Density plots of indegree, cumulative outdegree, sum cumulative cosine, and mean |log2FC| (Mean |FC|) for target genes. Red polygons represent the upper quartile for each parameter. Furthermore, 254 targets were in the upper quarter for all four parameters. (b) Heatmap depicting the log2FC (FC) of prioritized target genes across both interactions. (TIF) [file pone.0287590.s006.tif]
