# Supplementary material for: Gene regulatory network inference in soybean upon infection by Phytophthora sojae
Source: PLoS One. 2023 Jul 7;18(7):e0287590. doi: 10.1371/journal.pone.0287590 (PMC10328377; doi:10.1371/journal.pone.0287590)
Supplement: S1 Table — (PDF) [file pone.0287590.s007.pdf]

Table S1 RNA-seq mapping statistics

Sample name	M1	M2	M3	M6	R1_1	R1_2	R1_4	R1_6	R25_1	R25_2	R25_4	R25_5	R25_6
Total reads	38796124	47283140	40648056	49051920	45122968	39972182	43767076	41355640	45424202	46101140	43826198	41955178	43321070
Total mapped reads	37250753	45223757	38566918	46701795	43223395	37838386	41078577	39398714	43537969	44353091	41755041	39804441	40124980
Uniquely mapped reads	36335719	44190723	37756122	45416475	42261543	36845885	39927815	38480256	42465614	43377940	40722416	38784709	38808018
Multiple mapped reads	915034	1033034	810796	1285320	961852	992501	1150762	918458	1072355	975151	1032625	1019732	1316962
Total mapping rate	96.02%	95.64%	94.88%	95.21%	95.79%	94.66%	93.86%	95.27%	95.85%	96.21%	95.27%	94.87%	92.62%
Uniquely mapping rate	93.66%	93.46%	92.89%	92.59%	93.66%	92.18%	91.23%	93.05%	93.49%	94.09%	92.92%	92.44%	89.58%
Multiple mapping rate	2.36%	2.18%	1.99%	2.62%	2.13%	2.48%	2.63%	2.22%	2.36%	2.12%	2.36%	2.43%	3.04%
