# Supplementary material for: Gene regulatory network inference in soybean upon infection by Phytophthora sojae
Source: PLoS One. 2023 Jul 7;18(7):e0287590. doi: 10.1371/journal.pone.0287590 (PMC10328377; doi:10.1371/journal.pone.0287590)
Supplement: S3 Table — (PDF) [file pone.0287590.s009.pdf]

Table S3 DAP- and AmpDAP-seq mapping statistics

	Total reads	Unique mapped reads	Unique mapping rate
**WRKY30_M1**	12914375	2587348	20.0
**WRKY30_P1**	14475563	8044238	55.6
**GmMYB61**	22912058	21655567	94.5
**GmWRKY2**	18347627	16694981	91.0
**Mock treatment (Empty Vector - CD3-1742)**	19679731	2209370	11.2
**Pathogen treatment (Empty Vector - CD3-1742)**	17970979	1830852	10.2
**SRR13197372 (GmRAV rep1)**	16884373	12931211	76.6
**SRR13197373 (GmRAV rep2)**	16512952	12625327	76.5
**SRR13197374 (background control)**	54817690	45746987	83.5
