# Supplementary material for: Gene regulatory network inference in soybean upon infection by Phytophthora sojae
Source: PLoS One. 2023 Jul 7;18(7):e0287590. doi: 10.1371/journal.pone.0287590 (PMC10328377; doi:10.1371/journal.pone.0287590)
Supplement: S1 File — (PDF) [file pone.0287590.s017.pdf]

Gene regulatory network inference in soybean upon infection by *Phytophthora sojae*

Brett Hale[1,2,3,¶], Sandaruwan Ratnayake[2,3,¶,#a], Ashley Flory[2,#b], Ravindu Wijeratne[4], Clarice Schmidt[5], Alison E. Robertson[5], and Asela J. Wijeratne[2,3,*]

[1]Molecular Biosciences Graduate Program, Arkansas State University, State University, AR, USA

[2]Arkansas Biosciences Institute, Arkansas State University, State University, AR, USA

[3]College of Science and Mathematics, Arkansas State University, State University, AR, USA

[4]Houston High School, Germantown, TN, USA

[5]Department of Plant Pathology and Microbiology, Iowa State University, Ames, IA, USA

[¶]These authors contributed equally to this work.

[#a]Current address: Donald Danforth Plant Science Center, St. Louis, MO, USA

[#b]Current address: St. Jude Children's Research Hospital, Memphis, TN, USA

*Corresponding author
E-mail: awijeratne@astate.edu

SUPPORTING INFORMATION

Fig S1 Capture-seq validation of RNA-seq data

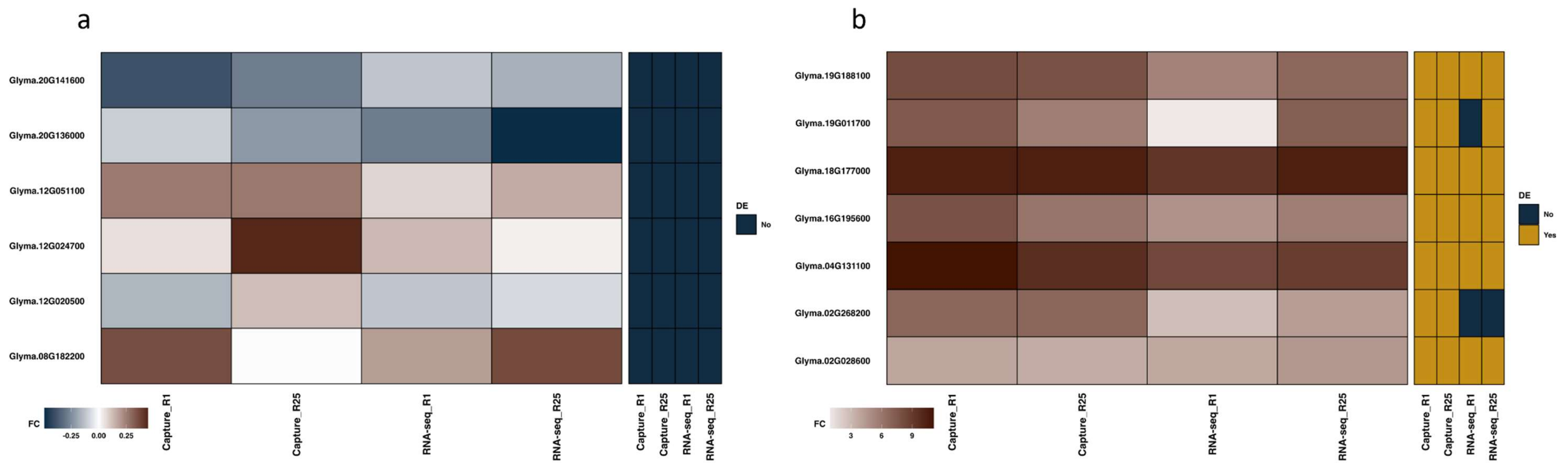

Fig S2 Selection of 201-bp bound (peak regions) and unbound sites (negative dataset) during model training

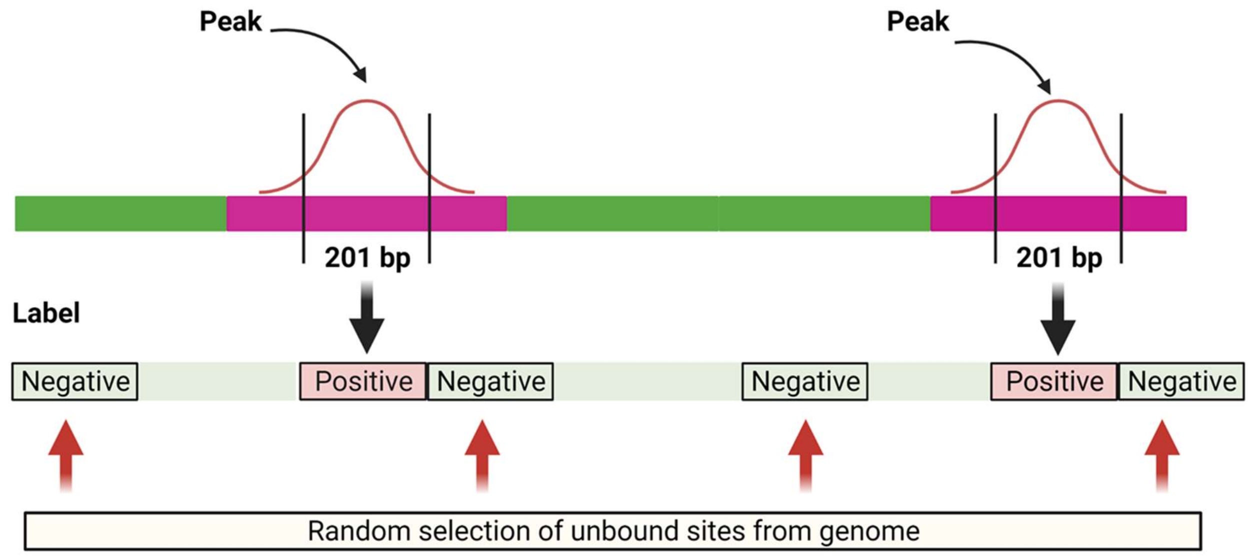

Fig S3 auROC and auPRC curves for soybean data-trained models

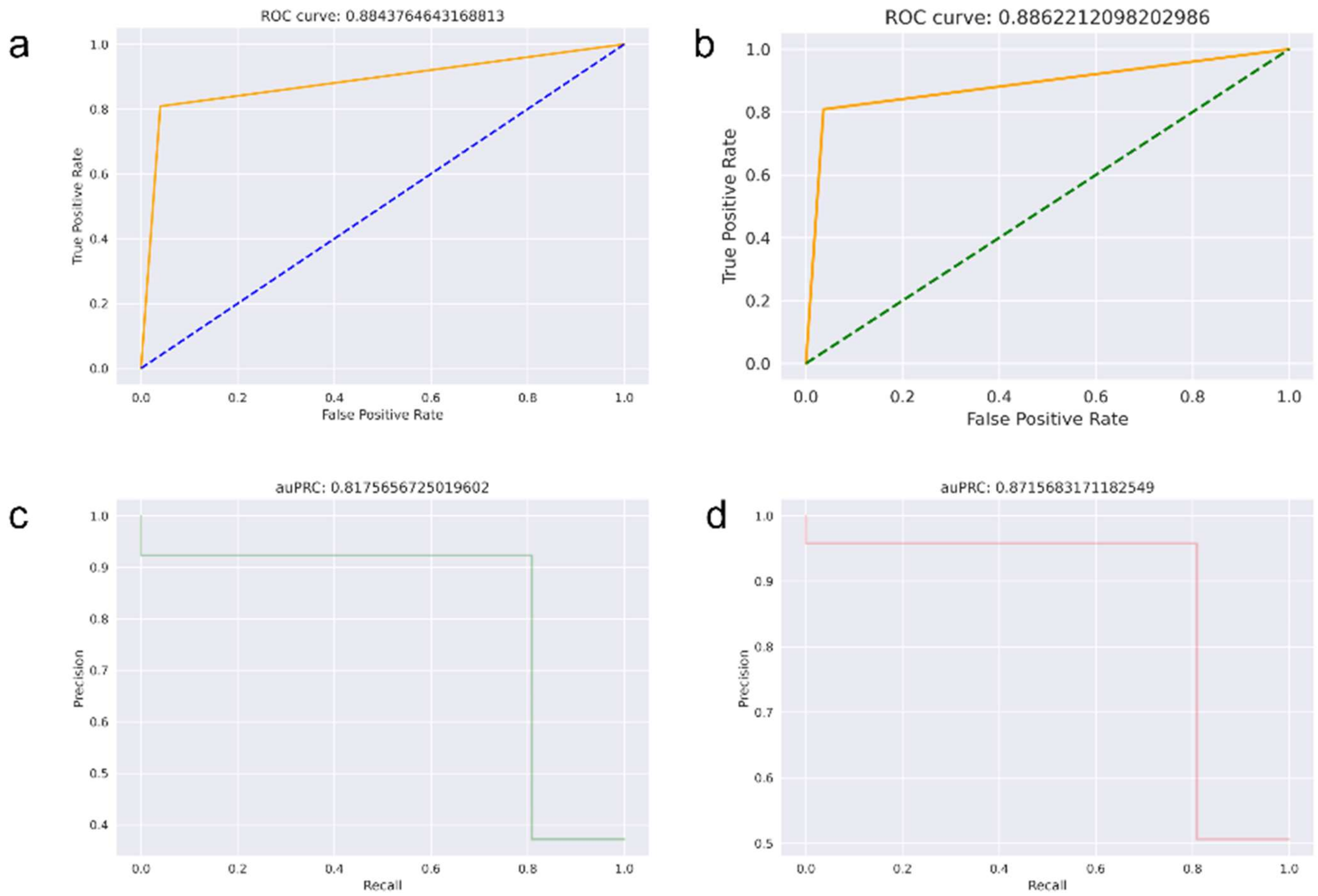

Fig S4 AmpDAP-seq data for GmMYB61 and GmWRKY2

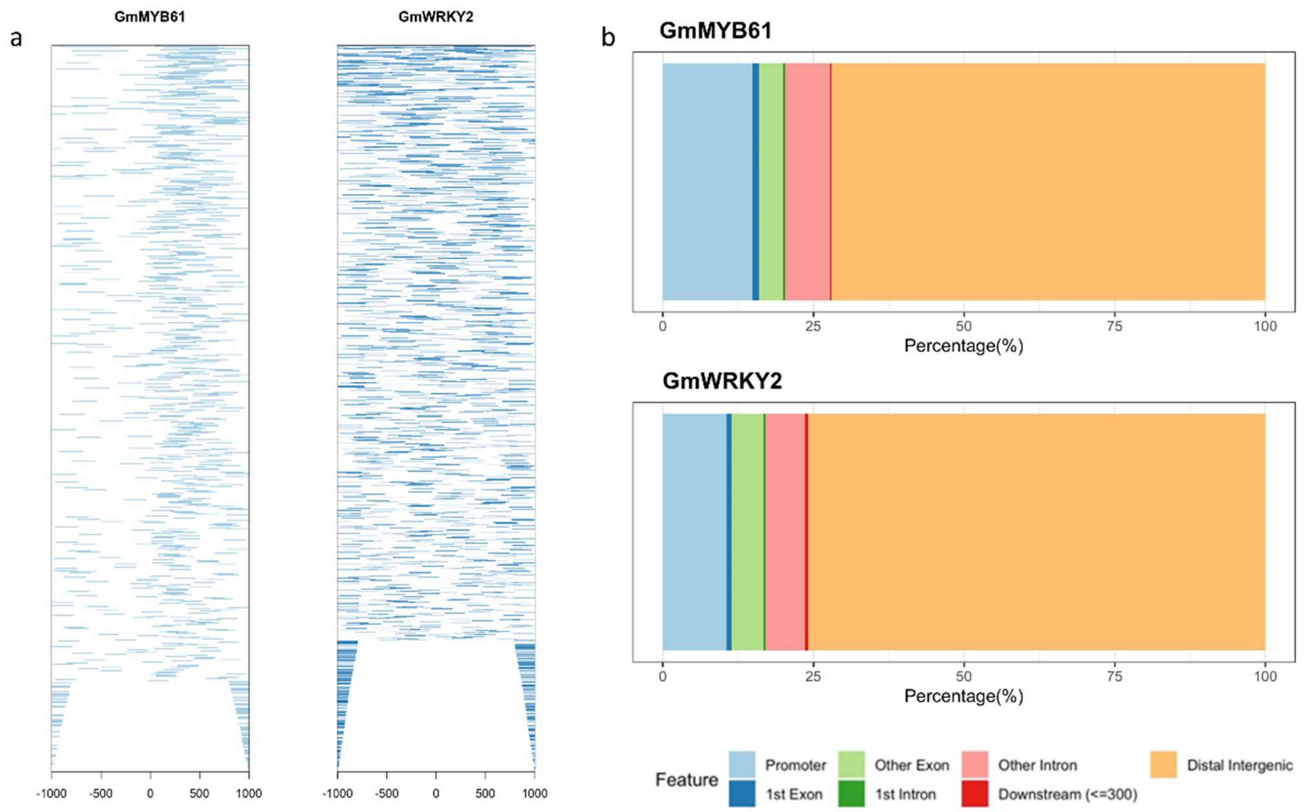

Fig S5 GRNs for defense-related TF families

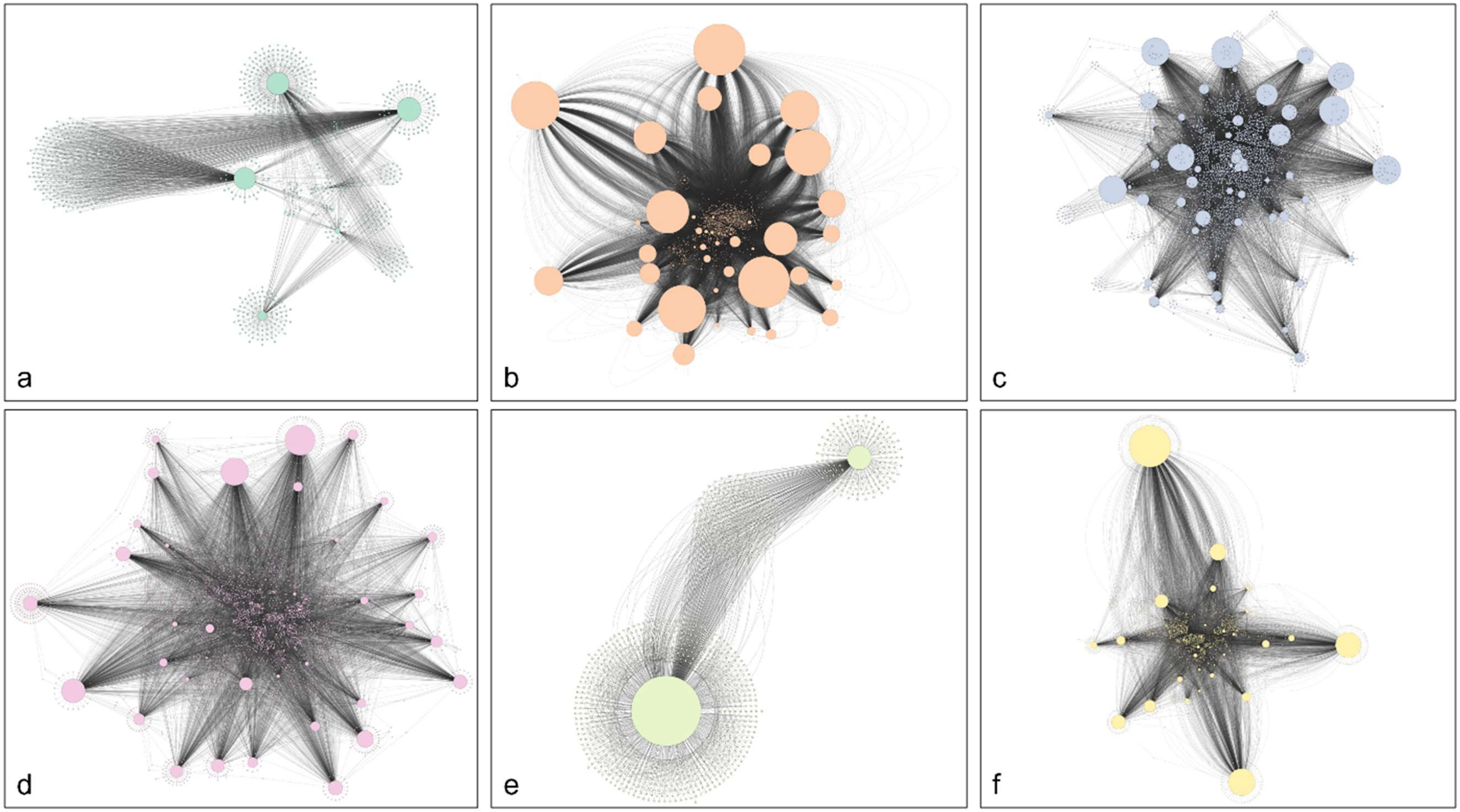

Figure S6 Prioritization of target genes

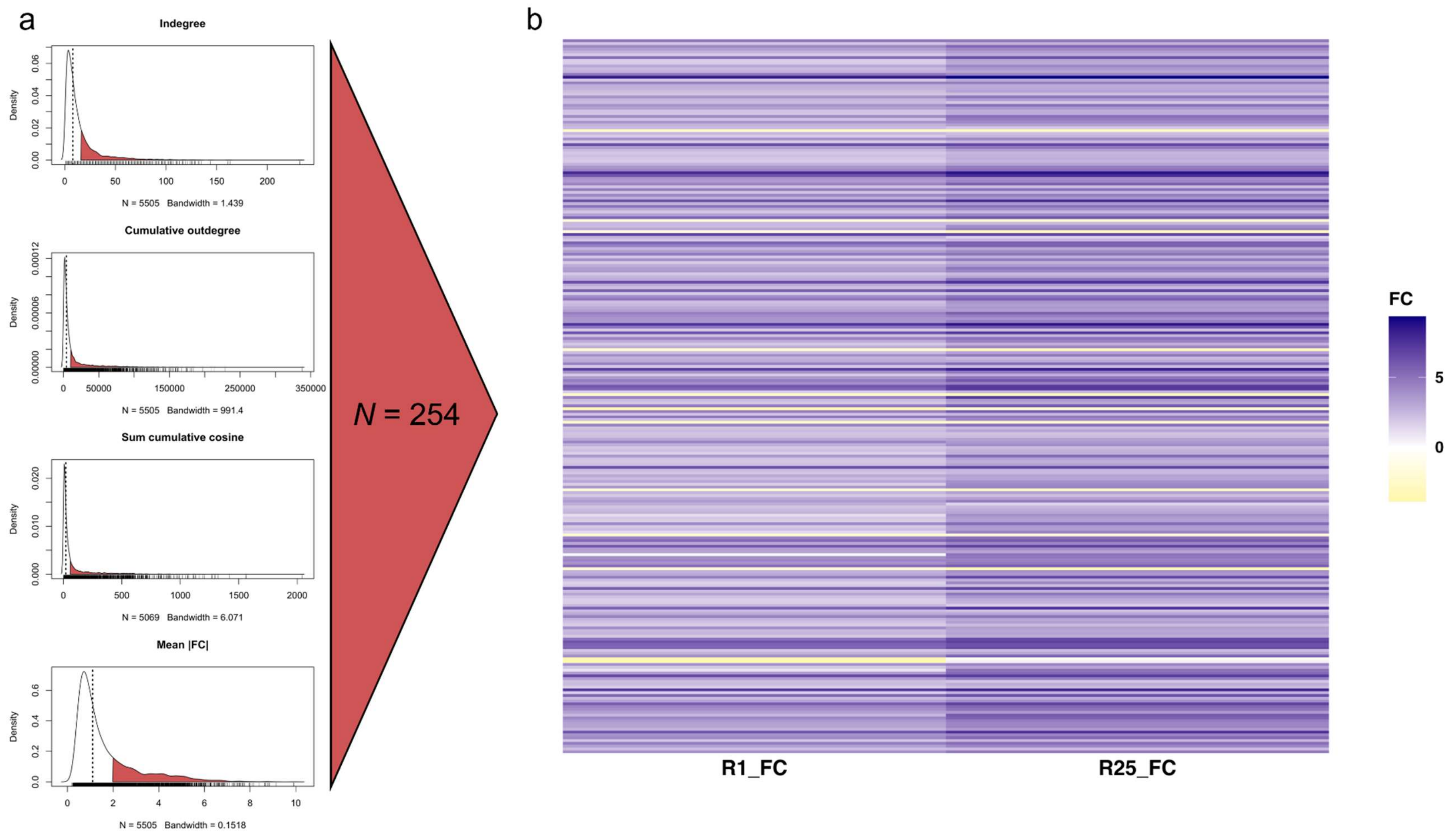

Table S1 RNA-seq mapping statistics

Sample name	M1	M2	M3	M6	R1_1	R1_2	R1_4	R1_6	R25_1	R25_2	R25_4	R25_5	R25_6
**Total reads**	38796124	47283140	40648056	49051920	45122968	39972182	43767076	41355640	45424202	46101140	43826198	41955178	43321070
**Total mapped reads**	37250753	45223757	38566918	46701795	43223395	37838386	41078577	39398714	43537969	44353091	41755041	39804441	40124980
**Uniquely mapped reads**	36335719	44190723	37756122	45416475	42261543	36845885	39927815	38480256	42465614	43377940	40722416	38784709	38808018
**Multiple mapped reads**	915034	1033034	810796	1285320	961852	992501	1150762	918458	1072355	975151	1032625	1019732	1316962
**Total mapping rate**	96.02	95.64	94.88	95.21	95.79	94.66	93.86	95.27	95.85	96.21	95.27	94.87	92.62
**Uniquely mapping rate**	93.66	93.46	92.89	92.59	93.66	92.18	91.23	93.05	93.49	94.09	92.92	92.44	89.58
**Multiple mapping rate**	2.36	2.18	1.99	2.62	2.13	2.48	2.63	2.22	2.36	2.12	2.36	2.43	3.04

Table S2 Capture-seq mapping statistics

	TC_1_1	TC_1_2	TC_1_3	TC_1_4	TC_1_5	TC_1_6	TC_1_7	TC_1_8
**Processed reads**	1135014	1037646	1364621	757173	472378	594797	1067169	642834
**Pseudoaligned reads**	1106470	1011519	1329715	737622	460848	580083	1044934	627582
**Unique reads**	1099544	1003162	1321370	736926	459854	579011	1035882	624194
**Pseudoaligned read mapping rate**	97.5	97.5	97.4	97.4	97.6	97.5	97.9	97.6
**Unique read mapping rate**	96.9	96.7	96.8	97.3	97.3	97.3	97.1	97.1

Table S3 DAP- and AmpDAP-seq mapping statistics

	Total reads	Unique mapped reads	Unique mapping rate
**WRKY30_M1**	12914375	2587348	20.0
**WRKY30_P1**	14475563	8044238	55.6
**GmMYB61**	22912058	21655567	94.5
**GmWRKY2**	18347627	16694981	91.0
**Mock treatment (Empty Vector - CD3-1742)**	19679731	2209370	11.2
**Pathogen treatment (Empty Vector - CD3-1742)**	17970979	1830852	10.2
**SRR13197372 (GmRAV rep1)**	16884373	12931211	76.6
**SRR13197373 (GmRAV rep2)**	16512952	12625327	76.5
**SRR13197374 (background control)**	54817690	45746987	83.5
